# Analysis of hand-forearm anthropometric components in assessing handgrip and pinch strengths of school-aged children and adolescents: a partial least squares (PLS) approach

**DOI:** 10.1186/s12887-020-02468-0

**Published:** 2021-01-15

**Authors:** Sajjad Rostamzadeh, Mahnaz Saremi, Shahram Vosoughi, Bruce Bradtmiller, Leila Janani, Ali Asghar Farshad, Fereshteh Taheri

**Affiliations:** 1 Occupational Health Research Center, Iran University of Medical Sciences, Tehran, Iran; 2 Workplace Health Promotion Research Center, School of Public Health and Safety, Shahid Beheshti University of Medical Sciences, Tehran, Iran; 3 Department of Occupational Health Engineering, Occupational Health Research Center, Faculty of Health, Iran University of Medical Sciences, Shahid Hemmat Highway, Tehran, Iran; 4 Anthrotech, Inc, OH Yellow Springs, USA; 5 Department of Biostatistics, School of Public Health, Iran University of Medical Sciences, Tehran, Iran

**Keywords:** Handgrip, Pinch, Partial Least Squares (PLS), Adolescents, Anthropometry

## Abstract

**Background:**

The purpose of this study was to examine the influence of hand-forearm anthropometric dimensions on handgrip and pinch strengths among 7–18 years children and adolescents and to investigate the extent to which these variables can be used to predict hand strength.

**Methods:**

Four types of hand strengths including handgrip, tip to tip, key, and three-jaw chuck pinches were measured in 2637 healthy children and adolescents (1391 boys and 1246 girls) aged 7–18 years using standard adjustable Jamar hydraulic hand dynamometer and pinch gauge. A set of 17 hand-forearm anthropometric dimensions were also measured with an accurate digital caliper and tape measure.

**Results:**

No significant differences were found between the hand strengths of boys and girls up to the age of 10 years. Gender related differences in handgrip and pinches were observed from the age of 11 years onwards, with boys always being stronger. The dominant hand was stronger than the non-dominant hand (8% for handgrip and by about 10% for all three types of pinches). The strongest correlations were found between the hand length and hand strengths (r > 0.83 for handgrip and three all pinches; p < 0.001, 2-tailed). Based on the partial least squares (PLS) analysis, 8 out of 17 anthropometric indices including hand length, hand circumference, thumb length, index finger length, middle finger length, and forearm length had considerable loadings in the PLS analysis, which together accounted for 46% of the total variance.

**Conclusions:**

These results may be used by health professionals in clinical settings as well as by designers to create ergonomic hand tools.

## Background

Muscle strength is an important aspect of physical fitness, locomotor skills, nervous system maturation and health status in children’s development [1, 2]. Therefore, growth-related changes in the muscle strength of healthy children may be considered as a reference for children and adolescents with acute and chronic diseases [3, 4]. In children and adolescents, muscle strength has negatively related to the clustered metabolic risk independent of cardiorespiratory fitness [5, 6]. A low level of muscular strength in children and adolescents is associated with poorer metabolic profile, obesity, high blood pressure, all-cause premature, and mortality in adulthood [7, 8].

Handgrip strength (HGS) is a useful marker of physical strength throughout an individual’s lifetime and is often estimated in screenings of normal motor function [7]. Handgrip strength and pinch strength (PS) are determinative for performing prehensile and precision hand functions and daily muscular activities through the use of exquisitely arranged power and precision muscles, which acting through the extensor hood, work in synergistic precision to manipulate the digits [9, 10]. Considered as the most reliable clinical tests for measuring maximum isometric strength of hand-forearm muscles, the values of handgrip and pinch strengths determine the efficacy of different treatment strategies [3, 11]. These measures are often used as a functional index for nutritional status, insulin sensitivity, overall function of the upper limb, and cardio metabolic health [12, 13].

Age, gender, hand preference and anthropometric dimensions are among the most cited factors influencing handgrip and pinch forces [14]. Stronger handgrip and pinch strengths were reported for boys compared to girls, and for dominant hand compared to the non-dominant one [15, 16]. Moreover, handgrip and pinch strengths increases linearly with age in both boys and girls [17, 18]. More precisely, HGS and PSs start to grow from childhood and reach a maximum level at the age of 30 s to dwindle afterwards [19, 20]. Some studies have also shown that although height and weight are positively correlated with hand strengths in pubertal years, the influence of these variables is considerably smaller than that of either gender or age [17, 21]. Considering anthropometric dimensions, previous studies have found a strong correlation with handgrip and pinch strengths in adults [22–24], which was confirmed in children and adolescents in few studies [25]. In addition, there are contradictory findings in the studies on the anthropometric variables affecting hand strengths (HGS and PSs) in different age ranges [20, 26].

Due to a large number of variables affecting hand strengths (HGS and PSs), the presence of more than one response variable in the study as well as strong linear correlation among explanatory variables making it difficult to separate their effects on the dependent variable; engineering approaches are incapable of solving these problems. Several approaches have been introduced to address this issue, among which Partial Least Squares (PLS) is a good competitor [27]. As a multivariate method, PLS is strongly related to regression-like techniques which can be used as an exploratory analysis tool to select suitable predictor variables and to identify outliers before standard linear regression. More precisely, PLS predicts a set of dependent variables from a set of independent variables or predictors. Contrary to the standard regression which predicts one variable only, PLS is used to predict a whole table of data. These features make PLS a very versatile tool specified as a robust method, in which the model parameters do not significantly change if new samples are taken from the same population. PLS as a variance-based method is mainly used as an alternative for modelling structural equations, in contrast to older methods based on covariance [28].

To the best of the authors’ knowledge, only a limited number of studies have investigated the association between handgrip and pinch strengths with anthropometric dimensions among children and adolescents, particularly in Asia. Given the above, the present study was carried out to analyze the hand-forearm anthropometric components in assessing handgrip and pinch strengths in school-aged children and adolescents and to investigate if they can be used to predict these outcomes.

## Methods

### Participants and sampling

This cross-sectional study was conducted on 2637 school-aged children and adolescents (1391 boys and 1246 girls) between the age of 7 and 18 years from different districts of the major metropolitan city of Tehran, Iran. Data collection was carried out between February and May 2019.

The three-stage sampling method was utilized. At first, a stratified sampling method was used to identify 10 clusters based on population distribution in Tehran. In the second stage, after providing the list of all the schools located in selected clusters, a systematic random sampling method was applied to choose four schools per cluster (one elementary and one high school for each gender). The required minimum sample size at any of the girls’ or boys’ schools was estimated using Eq. (1) given in “General requirements for establishing anthropometric databases” [29]. The 95% confidence interval was used for the 50th percentile or average values:

1 $$n \geq (3.006 \times \frac{\text{C}\text{V}}{{\upalpha}})^2\ and\ CV= \frac{S}{\bar{X}} \times 100$$

In this formula, n, CV, and α represents the sample size, coefficient of variation and percentage of the desired relative accuracy, respectively. Assuming a relative accuracy of 5% and using the empirical means and standard deviations (boys: 22.8 kg and 2.9 kg with CV = 12.7; girls: 17.4 kg and 2.3 kg with CV = 13.2) from the results of the initial pilot study of 80 participants (40 for each gender), the minimum required sample sizes were calculated as 58 for boys and 63 for girls in each school. Considering the “Design effect” for clustered sampling method (Deff = 2.2) [30], the desired sample size was obtained 2637 subjects with about 10% non-response rate.

All students over 16 years and the parents/guardians of all minor participants (< 16 years) signed consent form describing the aims and procedures of this study. The principle of voluntary participation was respected. Using a short health screening questionnaire, students with history of fracture, deformity or surgery in upper extremities during the past year as well as those with history of specific diseases such as osteoarthritis, rheumatic arthritis, coronary heart disease, chronic obstructive pulmonary disease, sequelae after stroke, chronic kidney disease, and liver cirrhosis were excluded to ensure a healthy study sample. The impact of these diseases on upper extremities function, especially the arms and hands, has been shown in previous studies [31, 32]. The study was conducted according to the World Medical Association Declaration of Helsinki and was approved by the ethics committee, Iran University of medical science (IR.IUMS.REC 1396.32516).

### Measurements

One trained examiner was recruited per study outcomes (i.e. one for anthropometric measurements and one for handgrip and pinch strengths measurements). Therefore, measurements of the same types were obtained by the same examiner for all students during the study period. All measurements were obtained in a separate room dedicated to the school health supervisor during the school day from 8 to 12 AM.

### Anthropometric measurements

Age of participants was recorded from their academic records. Body mass was measured to the nearest 0.1 kg by a digital balance (Toledo, Model 2096PP/2, Inc., Brazil). Stature was measured for each subject using the Holtain Harpenden stadiometer (Holtain, Crosswell, UK). Body mass index was calculated in kg/m^2^.

**Fig. 1:**
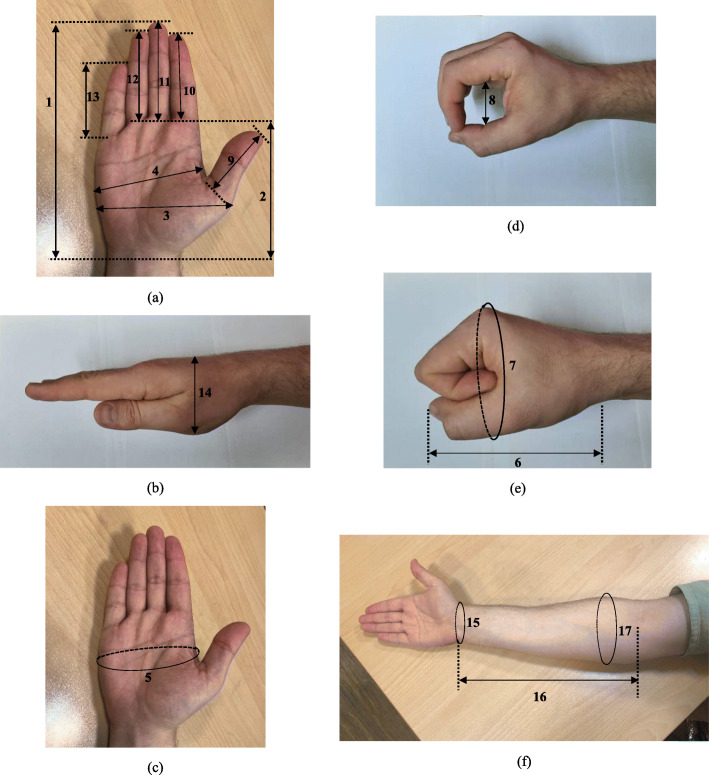
Hand-forearm anthropometric relative landmarks

A set of 17 hand-forearm anthropometric dimensions were measured for each student. Definitions and methods used for measurement correspond to the ISO7250-1:2017 [33]. Description of anthropometric measurements as well as their relative landmarks are presented in Table 1; Fig. 1, respectively. Garrett et al. (1971) showed that wrist crease is the best landmark for easy identification of hand dimensions. Thus, the right hand is held out horizontally such that the palm faced upwards and the fingers are extended. When whole measurements are to be taken, the fingers are kept close together (adducted) to measure individual fingers length [34]. These hand dimensions were measured with an accurate JEGS digital caliper (Model: 80,519, Columbus, OH 43,211, USA; ±0.01 mm) and a tape measure (HaB Essentials SKU: LCR01; ±0.1 cm). Measurements were repeated twice for each hand. The average of the two values for each dimension was calculated and recorded for analysis. All participants were wearing light clothing during measurements and were asked to remove heavy outer garments and jewelry. Also, proper care has been taken to avoid any excessive compression of the underlying tissues and to record the measurement precisely during the measurement.

**Table 1:** Description of hand-forearm dimensions measured in the study

Hand-forearm dimension	Definitions
1) Hand length	• The distance from the base of the hand to the top of the middle finger measured along the long axis of the hand
2) Palm length	• The distance from the base of the palm to the base of the middle finger (at the palmar surface).
3) Hand breadth across thumb	• The breadth of the hand measured at the level of the distal end of the first metacarpal of the thumb.
4) Hand breadth metacarpal	• The breadth of the hand as measured across the distal ends of the metacarpal bones.
5) Hand circumference	• The close measurement that follows a hand contour at the maximum palm level.
6) Fist length	• Length of the hand grip in the same line of the long axis of the hand from the base of the palm to the tip of the fist, wherever found.
7) Fist circumference	• Maximum circumference of the fist, wherever found, encompassing the knuckles of the middle finger and the thumb.
8) Maximum internal grip diameter	• The measured by sliding the hand down a graduated cone until the tips of the thumb and the middle finger remain touched to each other
9) Thumb length (digit 1)	• The distance from the tip of the thumb to its proximal crease.
10) Index finger length (digit 2)	• The distance from the tip of the index finger to its proximal crease.
11) Middle finger length (digit 3)	• The distance from the tip of the middle finger to its proximal crease.
12) Ring finger length (digit 4)	• The distance from the tip of the ring finger to its proximal crease.
13) Pinky finger length (digit 5)	• The distance from the tip of the pinky finger to its proximal crease.
14) Hand depth	• The maximum depth from the volar side of the thenar pad to the dorsal surface of the hand (Hand is extended with palm facing down; fingers are close together with the thumb held against the side of the hand)
15) Wrist circumference	• This dimension is measured using a tape measure, which is wrapped around the bony part of wrist, snug but not tight.
16) Forearm Length	• The distance between radial styloid process and lateral humeral epicondyle.
17) Forearm Circumference	• The circumference of the forearm at the point of maximum prominence, slightly distal to the elbow joint.

### HGS and PSs Measurements

Handgrip and PSs (tip to tip, key, and three-jaw chuck pinches) strengths were measured with a Jamar hydraulic hand dynamometer and pinch gauge (Model 5030J1, Sammons Preston Rolyan, Bolingbrook, IL, USA) according to the recommendation of the American Society of Hand Therapists (ASHT) [35]. Jamar dynamometer is recommended as the gold standard by the ASHT, leading to its extensive use in clinical practice and research. In most studies, the authors investigated the reliability and reproducibility of Jamar hand-held dynamometer for children/adolescents in general [36, 37]. For standardization, the dynamometer was set at the second handle position for measurement of handgrip strength [11]. Before starting the test, hand dominance was determined by asking participants the following question: “Which hand do you write with? “. Handgrip and then pinch strengths were measured while students were seated with feet on the floor, arms hanging relaxed at the side and neutrally rotated, elbows flexed 90 degrees, and forearm and wrist in neutral position (0–15 degrees of extension and 0–15 degrees of ulnar deviation) [38]. In all the cases, the forearm and arm were not supported by the examiner or by an armrest. Students were asked to squeeze the handle of the dynamometer as well as the pinch gauge button as hard as they can and to sustain the effort for 3 seconds. One-minute rests were given between each attempt to minimize fatigue affects. Verbal encouragement was provided to ensure maximal effort during each test. The readings were recorded from three trials for each hand, and the average of the three values was considered as the HGS and PSs (tip to tip, key, and three-jaw chuck pinches) values for subsequent analyses. If one of the measurements had a difference higher than 10% compared to other measurements, it was cancelled and replaced by a forth measurement. These procedures have been previously well documented as reliable [39, 40]. The calibration of instruments was tested periodically during the study according to the manufacturer’s manual. The dynamometer and pinch gauge were set to zero kg before each measurement.

### Data analysis

Statistical analysis was performed by SPSS 23 (IBM Corporation, New York, NY, United States). In the beginning, normality test was carried out using the one-sample Kolmogorov-Smirnov (K-S) test and confirmed for all data sets. Statistical outliers were checked using Grubb′s test that is based on the difference of the mean of the sample and the most extreme data considering the standard deviation [41]. Test-retest reliability was analyzed using interclass correlation coefficient (ICC). Independent sample t-test was carried out to determine the HGS and PSs differences between boys and girls students. Paired t-tests were performed to compare the handgrip and pinch (tip to tip, key, and three-jaw chuck) strengths of both hands (dominant vs. non-dominant hand). One-way ANOVA test was used to compare dominant and non-dominant hand strengths (HGS and PSs), allocated according to age groups and gender. The Tukey post-hoc test in separate analyses of variances was used to examine differences between specific age groups for both genders. Pearson’s correlation coefficient test was used to determine the correlations of the hand-forearm anthropometric and demographic variables with HGS and three pinches values.

SMART-PLS 3.0 software was additionally used to determine the possible correlations between anthropometric/demographic measures and hand strengths (HGS and PSs) outcomes in a multivariate approach, which has advantages over regression-based methods in evaluating several independent (manifest) variables with various dependent (latent) variables [42]. This fact underlines that the most essential part of a PLS analysis is the estimation of the weight relations. Of course, it would be easier simply to assume equal weights for all variables, but this approach has two disadvantages: First, there is no theoretical rationale for all indicators to have the same weighting. Because it can be assumed that the resulting parameter estimates of the structural model depend on the type of weighting used, at least as long as the number of indicators is not excessively large [43]. Second, as Chin et al. (2003) stressed, such a procedure does not take into account the fact that some indicators may be more reliable than others and should, therefore, receive higher weights [44]. For this purpose, four different types of hand strengths (HGS and tip to tip, key, and three-jaw chuck pinches) were considered as dependent variables, while independent variables were hand-forearm anthropometric measures. Based on the PLS method, items which had a factor loading greater than 0.25 were selected as the most important variables to explain the majority of the total variance of the model [45]. The significance level was set at 0.05.

## Results

### ***Demographic and hand-forearm anthropometric data***

Demographic information including age, gender, hand dominance, and hand-forearm anthropometric characteristics of the study participants are shown in Tables 2 and 3. The sample consisted of 2637 healthy children and adolescents students-aged 7–18 years including 1391 (52.7%) boys and 1246 (47.3%) girls. Right-hand dominance was reported by 2506 (95%) students comprising 1319 (50%) boys and 1187 (45%) girls. None of the students reported ambidexterity.

Results of test-retest reliability were analyzed from 80 participants out of whole sample. Participants showed high to very high test-retest reliability for Jamar dynamometer (0.84 ≤ ICC ≤ 0.96; *P* ≤ 0.001) and pinch gauge (0.86 ≤ ICC ≤ 0.92; *P* ≤ 0.001).

**Table 2:** Characteristics of study participants: age, gender, and hand dominance

Age (years)	N	Boys			Girls		
		n	Dominant Hand		n	Dominant Hand	
			HandRight	Left		Right	Left
7	230	126	121	5	104	100	4
8	213	110	103	7	103	98	5
9	235	125	121	4	110	105	5
10	223	116	111	5	107	100	7
11	208	108	105	3	100	96	4
12	212	116	110	6	96	92	4
13	218	114	107	7	104	103	1
14	218	107	103	4	111	105	6
15	208	109	102	7	99	95	4
16	226	118	110	8	108	104	4
17	224	120	111	9	104	96	8
18	222	122	115	7	100	93	7
**Total**	**2637**	**1391**	**1319**	**72**	**1246**	**1187**	**59**

*N* number of participants per age group; *n* number of participants per gender

**Table 3:** Descriptive statistics of demographic and hand-forearm anthropometric variables

Variable	Boys (***n*** = 1391)		Girls (***n*** = 1246)	
	Mean ± SD	Min-Max	Mean ± SD	Min-Max
Age (years)	13.2 ± 3.7	7–18	12.6 ± 2.9	7–18
Stature (cm)	158.1 ± 9.1	108–193	148.8 ± 8.6	106–176
Weight (kg)	54.4 ± 8.5	24.1–84.3	44.8 ± 7.1	23.4–77.2
BMI	23.5 ± 9.0	18.8–34.8	22.1 ± 8.6	17.5–30.2
Hand length (cm)	17.6 ± 1.1	11.8–21.6	15.8 ± 0.9	10.0-20.5
Palm length (cm)	9.5 ± 0.8	7.1–12.0	8.9 ± 0.7	6.4–11.5
Hand breadth across thumb (cm)	8.6 ± 0.66	6.7–10.3	8.1 ± 0.60	6.3–9.3
Hand breath metacarpal (cm)	7.1 ± 0.51	5.4–8.1	6.5 ± 0.46	5.1–7.4
Hand circumference (cm)	19.5 ± 1.9	14.7–23.4	17.6 ± 1.6	14.1–21.3
Fist length (cm)	8.3 ± 0.68	6.4–10.2	7.9 ± 0.56	5.9–9.5
Fist circumference (cm)	22.4 ± 1.5	13.5–27.2	20.3 ± 1.3	13.2–25.6
Maximum Internal grip diameter (cm)	3.3 ± 0.55	1.8–4.6	3.0 ± 0.49	1.7-4.0
Thumb length (digit 1) (cm)	5.2 ± 0.49	4.1–6.3	4.8 ± 0.53	4.0-5.8
Index finger length (digit 2) (cm)	6.4 ± 0.43	5.3–7.8	5.9 ± 0.46	5.1–7.2
Middle finger length (digit 3) (cm)	7.0 ± 0.46	5.8–8.4	6.5 ± 0.47	5.6-8.0
Ring finger length (digit 4) (cm)	6.6 ± 0.51	4.8–7.5	6.0 ± 0.42	4.6–6.9
Pinky finger length (digit 5) (cm)	5.4 ± 0.48	4.2–6.5	4.9 ± 0.46	4.1-6.0
Hand depth (cm)	4.2 ± 0.40	2.8–5.3	3.9 ± 0.30	2.5–4.8
Wrist circumference (cm)	15.2 ± 1.3	10.9–19.8	14.4 ± 1.50	10.4–18.7
Forearm Length (cm)	22.1 ± 1.6	17.8–26.9	20.2 ± 0.48	17.0-24.8
Forearm Circumference (cm)	23.2 ± 1.4	14.5–29.6	21.0 ± 1.60	14.0-27.4

Table 4 shows the mean values for HGS, tip to tip, key, and three-jaw chuck pinches of the study population by gender, age group, and hand dominance. The ANOVA results showed significantly different levels of HGS and PSs outcomes in terms of the age group of participants in both genders (p < 0.05). According to the Tukey’s post hoc tests, boys and girls students in any age group exerted significantly higher levels of HGS as well as tip to tip, key, and three-jaw chuck pinches compared to their predecessor age group (p < 0.01). Boys of 11–14 and 15–18 years were stronger and had greater handgrip and pinch strengths than their girls peer groups (p < 0.001). Grip and pinch outcomes were marginally higher in 7–10 years boys compared to the girls of the same age range, but the differences were not statistically significant. More precisely, the average of girls’ HGS in 7–10, 11–14, and 15–18 years age groups were approximately 84%, 79%, and 60% of boys. Also, the average of girls’ tip to tip, key, and three-jaw chuck pinches were approximately 84.4%, 82.2%, and 88.5% of boys, respectively. Among different types of pinch, the key pinch produced the greatest strength followed by the tip to tip and three-jaw chuck pinches, whatever the gender and hand dominance.

Hand dominance had a significant effect on HGS and PSs outcomes (p < 0.001). The dominant HGS was greater than that of the non-dominant HGS by about 8% for both genders. Further, tip to tip, key, and three-jaw chuck pinches were significantly higher for the dominant, vs. non-dominant hand (about 9.3%, 10.5%, and 11.1% within boys and about 11.1%, 10.9%, and 10.4% within girls, respectively). The dominant hand tip to tip, key, and three-jaw chuck pinches exerted by boys’ students were 17.5%, 21.3%, and 13.2% higher than those exerted by girls, respectively. These values for the non-dominant hand tip to tip, key, and three-jaw chuck pinches exerted by boys’ students were 19.4%, 21.8%, 12.5% higher than of those exerted by girls, respectively.

**Table 4:** Differences in HGS and PSs measurements (kg) in terms of the hand dominance among genders, and age groups, where by Mean ± SD (Min-Max)

Age group (years)	Number	Hand	HGS	Tip to tip	Key	three-jaw chuck
Boys						
7–10	451	D	12.8 ± 2.6 (7.1–19.3)	3.3 ± 0.8 (1.6–4.8)	5.1 ± 1.0 (3.2–6.7)	4.1 ± 0.8 (1.8–6.4)
		ND	11.7 ± 2.4 (6.6–18.7)	3.1 ± 0.8 (1.5–4.2)	4.7 ± 0.9 (3.3–6.5)	3.7 ± 0.9 (1.9–5.7)
11–14	435	D	24.1 ± 4.2 (13.4–37.0)	4.5 ± 1.0 (2.8–6.1)	7.3 ± 1.0 (5.0–9.2)	6.1 ± 1.1 (3.2–8.4)
		ND	22.4 ± 4.2 (13.0–33.7)	4.1 ± 1.2 (2.7–5.8)	6.6 ± 1.2 (4.3–9.0)	5.4 ± 1.2 (3.0–7.8)
15–18	505	D	39.5 ± 4.0 (29.3–48.7)	6.2 ± 1.5 (3.9–8.1)	9.8 ± 1.5 (7.1–11.3)	8.0 ± 1.4 (4.8–10.1)
		ND	36.8 ± 4.2 (27.3–45.1)	5.6 ± 1.4 (3.1–7.6)	8.7 ± 1.4 (7.3–10.2)	7.1 ± 1.4 (5.0–9.7)
**Total**	**1391**	**D**	**25.5 ± 3.6 (7.1–48.7)**	**4.7 ± 1.0 (1.6–8.1)**	**7.4 ± 1.1 (3.2–11.3)**	**6.0 ± 1.1 (1.8–10.1)**
		**ND**	**23.6 ± 3.3 (6.6–45.1)**	**4.3 ± 1.1 (1.5–7.6)**	**6.7 ± 1.2 (3.3–10.2)**	**5.4 ± 1.0 (1.9–9.7)**
Girls						
7–10	357	D	11.3 ± 2.7 (6.1–18.4)	3.1 ± 0.8 (1.4–4.1)	4.6 ± 1.0 (3.0–6.2)	3.9 ± 1.0 (1.8–5.6)
		ND	10.2 ± 2.9 (5.3–17.8)	2.8 ± 0.9 (1.3–3.7)	4.1 ± 0.9 (2.9–5.8)	3.5 ± 0.9 (1.9–5.3)
11–14	421	D	18.9 ± 3.7 (12.7–27.5)	4.1 ± 1.0 (2.5–5.6)	6.3 ± 1.2 (4.3–8.8)	5.5 ± 1.1 (3.8–7.7)
		ND	17.8 ± 3.4 (13.0–29.2)	3.7 ± 1.1 (2.6–5.2)	5.7 ± 1.1 (4.5–8.0)	4.9 ± 1.0 (3.2–8.0)
15–18	468	D	23.7 ± 3.3 (17.7–30.4)	4.8 ± 0.9 (2.9–6.3)	7.6 ± 1.2 (4.6–9.4)	6.5 ± 1.0 (4.3–9.1)
		ND	22.1 ± 3.4 (16.5–29.8)	4.3 ± 0.8 (2.6–6.0)	6.8 ± 1.3 (4.9–9.0)	5.8 ± 1.1 (3.9–8.6)
**Total**	**1246**	**D**	**17.8 ± 3.0 (6.1–30.4)**	**4.0 ± 0.9 (1.4–6.3)**	**6.1 ± 1.1 (3.0-9.4)**	**5.3 ± 1.0 (1.8–9.1)**
		**ND**	**16.5 ± 3.1 (5.3–29.8)**	**3.6 ± 0.9 (1.3-6.0)**	**5.5 ± 1.1 (2.9-9.0)**	**4.8 ± 1.0 (1.9–8.6)**

Data are Means ± SD (Minimum–Maximum)

### Correlation analysis

Table 5 shows the Pearson’s correlation coefficients of the study variables. It was found that the correlations between 17 hand-forearm anthropometric dimensions and demographic factors with HGS are statistically significantly different from zero except fist circumference (r = 0.032; judged at p < 0.05, 2-tailed) and hand depth (r = 0.108; judged at p < 0.05, 2-tailed). The strongest correlations were found between the hand length and all types of hand strengths (0.845, 0.876, 0.892, and 0.835 for handgrip, tip to tip, key, and three-jaw chuck pinches, respectively; p < 0.01, 2-tailed). This was followed by the correlations of the stature and forearm length with HGS and PSs measurements (with the correlation coefficients being generally above 0.7). Moreover, there were significant correlations between different pinch types with demographic variables and some hand-forearm dimensions (age, stature, weight, BMI, hand length, hand circumference, thumb length, index finger length, middle finger length, wrist circumference, forearm length, and forearm circumference).

**Table 5:** Pearson’s correlation coefficients among hand-forearm anthropometric and demographic variables with HGS

variables	HGS		Tip to tip		Key		three-jaw chuck	
	r	p	r	p	r	p	r	p
Age	0.697^**^	0.000	0.623^**^	0.000	0.630^**^	0.000	0.662^**^	0.000
Stature	0.787^**^	0.000	0.783^**^	0.000	0.765^**^	0.000	0.755^**^	0.000
Weight	0.351^**^	0.000	0.411^**^	0.000	0.428^**^	0.000	0.383^**^	0.000
BMI	0.335^**^	0.001	0.331^**^	0.001	0.316^**^	0.001	0.307^**^	0.001
Hand length	0.845^**^	0.000	0.876^**^	0.000	0.892^**^	0.000	0.835^**^	0.000
Palm length	0.363^**^	0.000	0.161	0.165	0.137	0.229	0.171	0.153
Hand breadth across thumb	0.552^**^	0.000	0.081	0.248	0.131	0.245	0.140	0.227
Hand breadth metacarpal	0.443^**^	0.000	0.154	0.176	0.117	0.277	0.139	0.230
Hand circumference	0.361^**^	0.000	0.317^**^	0.001	0.376^**^	0.000	0.351^**^	0.000
Fist length	0.301^**^	0.001	0.188	0.155	0.171	0.231	0.109	0.334
Fist circumference	0.032	0.361	0.047	0.310	0.127	0.246	0.097	0.360
Maximum Internal grip diameter	0.563^**^	0.000	0.117	0.293	0.145	0.209	0.107	0.333
Thumb length (digit 1)	0.582^**^	0.000	0.521^**^	0.000	0.672^**^	0.000	0.664^**^	0.000
Index finger length (digit 2)	0.475^**^	0.000	0.265^*^	0.010	0.247^*^	0.018	0.272^*^	0.009
Middle finger length (digit 3)	0.478^**^	0.000	0.320^**^	0.001	0.318^**^	0.001	0.302^**^	0.001
Ring finger length (digit 4)	0.245^*^	0.015	0.133	0.209	0.145	0.194	0.127	0.231
Pinky finger length (digit 5)	0.210^*^	0.025	0.143	0.220	0.165	0.151	0.176	0.142
Hand depth	0.108	0.331	0.122	0.282	0.100	0.353	0.087	0.378
Wrist circumference	0.393^**^	0.000	0.251^*^	0.012	0.237^*^	0.020	0.206^*^	0.030
Forearm Length	0.745^**^	0.000	0.771^**^	0.000	0.727^**^	0.000	0.707^**^	0.000
Forearm Circumference	0.433^**^	0.000	0.203^*^	0.030	0.241^*^	0.006	0.210^*^	0.023

^*^Correlation is significant at the 0.05 levels (2-tailed).

^**^Correlation is significant at the 0.01 levels (2-tailed).

Bold numbers are significant at the 0.01 or 0.05 levels (2-tailed).

### ***Prediction of HGS and PSs strengths***

**Fig. 2:**
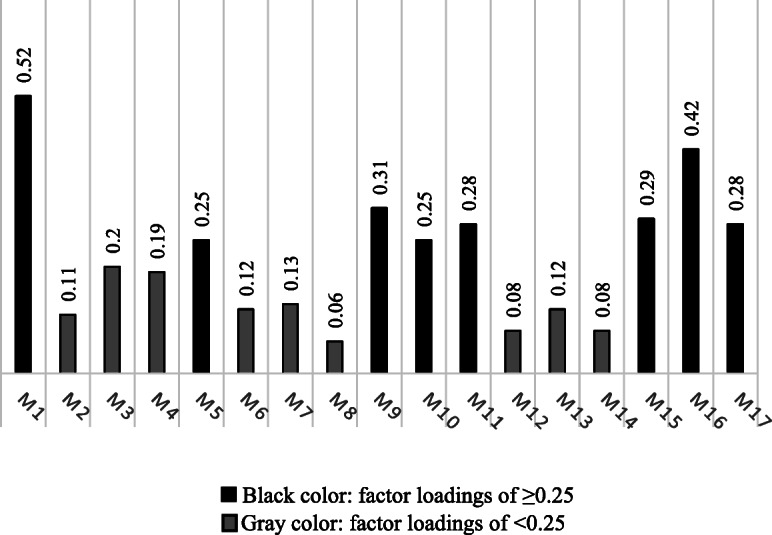
Factor loadings results from PLS for the independent (17 hand-forearm anthropometric) variables. Note: M1) Hand length; M2) Palm length; M3) Hand breadth across thumb; M4) Hand breath metacarpal; M5) Hand circumference; M6) Fist length; M7) Fist circumference; M8) Maximum Internal grip diameter; M9) Thumb length; M10) Index finger length; M11) Middle finger length; M12) Ring finger length; M13) Pinky finger length; M14) Hand depth; M15) Wrist circumference; M16) Forearm Length; M17) Forearm Circumference.

Based on the PLS analysis, only one factor was extracted which explained 46.14% and 58.81% of the total variance for the independent (hand-forearm dimensions) and dependent (handgrip and pinches) variables, respectively. The extracted dependent factor was correlated to the HGS, tip-to-tip pinch, and key pinch, three-jaw chuck pinch strengths, with the coefficients of 0.63, 0.51, 0.47 and 0.54, respectively. PLS factor loadings for the independent variables were compared in Fig. 2. Accordingly, hand length, hand circumference, thumb length, index finger length, middle finger length, forearm length had considerable factor loadings of > 0.25 in the extracted factor.

To estimate of internal consistency in the PLS approach, Cronbach’s $\alpha$ and item-delete Cronbach’s $\alpha$ are used for the extracted factor and each item, respectively (Table 6). The extracted factor had good internal consistency in the present study (The internal consistency is excellent if $\alpha \ge 0.9$, and good if 0.7$\le \alpha < 0.9)$ [46]. According to the results of the analysis, if items (M2), (M7), and (M14) are deleted, Cronbach’s $\alpha$ of the corresponding factor increases slightly.

**Table 6:** Internal consistency of factors

Cronbach’s ***a***	Item	Cronbach’s ***a*** if item is deleted
0.885	(M1) Hand length	**0.877**
	^a^(M2) Palm length	0.894
	(M5) Hand circumference	**0.861**
	^a^(M7) Fist circumference	0.888
	(M9) Thumb length (digit 1)	**0.875**
	(M10) Index finger length (digit 2)	**0.869**
	(M11) Middle finger length (digit 3)	**0.866**
	^a^(M14) Hand depth	0.891
	(M15) Wrist circumference	**0.875**
	(M16) Forearm Length	**0.862**
	(M17) Forearm Circumference	**0.879**

^a^Cronbach’s 훼 increases if item is deleted

Bold values indicate 0.7≤Cronbach’s α<0.9

## Discussion

Measurements of the handgrip and pinch strengths are convenient means to evaluate forearm and hand function. They can also be used to gauge the need for further physiotherapy during hand rehabilitation. In this study, the impact of hand-forearm anthropometric factors on handgrip and different three types of pinch strengths were examined among healthy children and adolescents aged 7–18 years. The extent to which hand-forearm anthropometric dimensions can be used to predict hand strengths was also investigated using a partial least squares (PLS) approach.

### Influence of age, gender, and handedness on hand strengths

We identified a linear increase in handgrip and pinch strengths of both hands associated with advancing age in boys and girls, in agreement with previous studies [47, 48]. This could be due to an exponential progression in muscle strength along with a rise in androgen hormones of both genders in pubertal years [49]. Considering gender and age, the present results showed almost similar handgrip and pinch strengths between boys and girls in 7–10 years age group when dominant or non-dominant hands were tested. This was consistent with the results of previous studies which found no differences in hand strengths between boys and girls until 10 years of age [48, 50]. Consistent with the review conducted by Omar et al. (2015), boys were stronger than girls, particularly after 11 years of age [51]. The onset of puberty, the period characterized by height gain and alterations in body composition, occurs about two years earlier in girls compared to boys. The body composition of boys and girls is similar before this period. During puberty, adipose deposition predominates in girls, whereas muscle mass increases in boys [52]. This divergence may explain the differences in hand strengths between genders after puberty. This hypothesis is supported by the fact that fat-free mass of muscle is one of the variables that has the greatest influence on handgrip and pinch strengths [53, 54]. Some studies also suggested that an increase in the testosterone of boys during puberty [55], muscle fiber type variability and neural adaptations in males [56], and higher usage of thenar muscles by males during sport and daily activities are known as factors for the gender-related differences [57].

The results of this study showed that hand dominance was a statistically significant factor in determining hand strength for the whole sample, a fact also reported in the literature [17, 47]. This is consistent with the findings of Sartorio et al. (2002) and Omar et al. (2018), which showed that the strength of the dominant hand was stronger than that of the non-dominant hand during puberty [18, 58]. It is interesting to note that the hand strengths differences between the dominant and non-dominant hands were generally similar in boys and girls studied. The present finding is consistent with previous study performed by Ng et al. (2019) who reported that there is almost similar strength difference between the dominant and non-dominant hands of boys and girls [59]. The percentage differences between the two sides can be calculated and used to determine the degree of rehabilitation required. Furthermore, hand strengths (HGS and PSs) were positively correlated with stature, weight, and BMI, where these results have been supported by Jurimaea et al. [12] and Rauch et al. [60]. These relationships may explain the higher hand strengths observed in the school-aged children with higher weight and BMI. Luna-Heredia et al. (2005), stated that body mass index (BMI) has a significant correlation with hand strength as an essential component of physical fitness, because it is the factor more closely related to body size [61]. The greater body size implies the development of long bones such as the ulna and radius, lengthening the arm-hand muscle fibers and thus developing new contractile units between the tendon and the muscle, facilitating the hand strength gain as a consequence [62]. On the other hand, higher BMI and excess adiposity can significantly affect different perspectives of pubertal development such as hormonal parameters during puberty and pubertal initiation time [63].

### Relationship between hand-forearm anthropometric and handgrip and pinch strengths

The present study reaffirms that hand size can be an important factor influencing handgrip and pinch strengths of children and adolescents. This finding is in more consistent with the results of Rostamzade et al. (2019), Boadella et al. (2005), and Maleki et al. (2019) studies, who found a positive correlation between hand length, hand breadth, forearm length and forearm circumference with hand strengths in healthy adults [23, 64, 65]. According to Rostamzadeh et al. (2020), some hand dimensions like hand breadth provides a greater contact area to apply more force to handle tools in grasping time [66]. They stated that strong and powerful movements during routine work-related activities increase the muscle mass in the upper extremities, especially the hand and forearm muscles, which can produce the higher hand strength in adulthood. It is documented that hand-forearm anthropometric measurements particularly palm width and forearm cicumference serve as better predictors of handgrip strength among adults than the more commonly recorded variables of weight and height [67]. Among the finger lengths that were measured, thumb length, index finger length, and middle finger length showed also strong correlations with all types of hand strength outcomes of both sides. Jurimӓe et al. (2009) suggested that the middle finger length is the most important hand dimension that influence handgrip strength in prepubertal children [12]. Studies conducted on adult subjects support this finding in the sense that index and middle finger lengths are strongly correlated with handgrip strength [62]. These findings were supported by the results of PLS analysis, which showed higher loading of these components compared to the others. However, it should be noted that in addition to these six anthropometric dimensions, there were two other anthropometric dimensions that had considerable loadings in the PLS analysis, which together accounted for 46% of total variance. One advantage of the PLS analysis is that it can be used as a method to identify more effective factors on dependent variables. In other words, when prediction is the goal and there is no practical need to limit the number of measured factors, PLS can be a useful tool [46]. Therefore, the results from this method provide a detailed understanding of the relationship between anthropometric traits and hand strengths (HGS and PSs). These findings can be used to establish predictive models for HGS and PSs, practical implications for the design of hand tools and stationeries, determine efficacy of rehabilitation, and assess the integrity of upper limb functions.

Our study reported the normative data of hand strengths (HGS and PSs) for Iranian children/adolescents. The reference values vary according to the races & ethnicities where the body structure and fat distribution varies considerably across different populations. Nevertheless, overall patterns of hand strength associated with advancing age in both genders were similar to findings of previous studies related to the progression of specific muscle strength in boys and girls living within different geographical areas [48, 68]. This reason can be expected to stem from several factors such as the mechanical stress leading to the increase of the body weight, the stretch imposed by growing long bones, a rise in androgen hormones of both genders in pubertal years, and possibly, the direct impact of adrenal and gender steroids on the muscle [49, 69]. The understanding of the behavior of hand strengths in the population is important to create parameters in physical rehabilitation programs, as well as for the exploration of grip and pinch force levels discriminating the risk of occurrence of health conditions. HGS values presented here as a reference may be employed in the clinical rehabilitation of upper limb function. They also serve as a global assessment component for children/adolescents individuals, especially in primary care, providing criteria for early identification of children’s with strength below the expected value. Such patients could be followed in order to be prevented from eventual future limitations or disabilities.

This study had a number of strengths. First, it was performed using a large sample of school-aged children and adolescents. Second, it used the standard protocols for handgrip and pinch strengths, hand-forearm anthropometric assessment as well as data monitoring processes during data collection, data entry and data analysis in order to minimize the risk of bias. To the best of the authors’ knowledge, no assessement is previously carried out to investigate the relationship of 17 hand-forearm anthropometric dimensions with handgrip and pinch strengths among children and adolescents in Asia. However, it should be noted that this study is somewhat limited because it was cross-sectional in design, which may potentially limit the generalizability of the results. In addition, some variables such as nutritional status, physical activity, and maturity stages were not considered, which may be a limitation due to the potential for misinterpretation.

## Conclusions

According to our findings, it can be concluded that ascending variability of the hand strengths (handgrip and pinches) in healthy children and adolescents can be explained by age, gender, handedness, and hand-forearm dimensions such as hand length and forearm length. A reference equation could be established based on these hand-forearm dimensions and demographic variables. These results can be used by health professionals in the clinical applications and hand rehabilitation as well as by designers to design hand tools and stationeries that should be balanced properly based on hand anthropometric dimensions. It can be argued that low hand strengths among children and adolescents warrants particular attention in order to identify the root cause, especially if there is no proportionality between the size of the hand tools and stationeries with the hand-forearm anthropometric dimensions.

## Data Availability

Data are not publicly available. These study data were not anonymous. Due to sensitive nature of the data and privacy and confidentially guidelines, the data must be housed in a secured lab and cannot be made publicly available.

## Abbreviations

PLS: Partial least squares
HGS: Handgrip strength
PSs: Pinch strengths
ICC: :Interclass correlation coefficient
D: Dominant
ND: Non-dominant

## References

[1] Takken T, Elst E, Spermon N, Helders PJM, Prakken ABJ, Van der Net J (2003). The physiological and physical determinants of functional ability measures in children with juvenile dermatomyositis. Rheumatology.

[2] Tanaka C, Hikihara Y, Ohkawara K, Tanaka S (2012). Locomotive and non-locomotive activity as determined by triaxial accelerometry and physical fitness in Japanese preschool children. Pediatr Exerc Sci.

[3] Wind AE, Takken T, Helders PJM, Engelbert RHH (2010). Is grip strength a predictor for total muscle strength in healthy children, adolescents, and young adults?. Eur J Pediatr.

[4] Windhager S, Schaefer K, Fink B (2011). Geometric morphometrics of male facial shape in relation to physical strength and perceived attractiveness, dominance, and masculinity. Am J Hum Biol.

[5] Buchan DS, Boddy LM, Young JD, Cooper S-M, Noakes TD, Mahoney C (2015). Relationships between cardiorespiratory and muscular fitness with cardiometabolic risk in adolescents. Res Sport Med.

[6] Morikawa SY, Fujihara K, Hatta M, Osawa T, Ishizawa M, Yamamoto M (2018). Relationships among cardiorespiratory fitness, muscular fitness, and cardiometabolic risk factors in Japanese adolescents: Niigata screening for and preventing the development of non-communicable disease study‐Agano (NICE EVIDENCE Study‐Agano) 2. Pediatr Diabetes.

[7] Ortega FB, Silventoinen K, Tynelius P, Rasmussen F (2012). Muscular strength in male adolescents and premature death: cohort study of one million participants. Bmj.

[8] Blakeley CE, Van Rompay MI, Schultz NS, Sacheck JM (2018). Relationship between muscle strength and dyslipidemia, serum 25 (OH) D, and weight status among diverse schoolchildren: a cross-sectional analysis. BMC Pediatr.

[9] Karakostis FA, Hotz G, Tourloukis V, Harvati K (2018). Evidence for precision grasping in Neandertal daily activities. Sci Adv.

[10] Ficuciello F (2019). Hand-arm autonomous grasping: Synergistic motions to enhance the learning process. Intell Serv Robot.

[11] Musa TH, Li W, Xiaoshan L, Guo Y, Wenjuan Y, Xuan Y (2018). Association of normative values of grip strength with anthropometric variables among students, in Jiangsu Province. HOMO.

[12] Jürimäe T, Hurbo T, Jürimäe J (2009). Relationship of handgrip strength with anthropometric and body composition variables in prepubertal children. HOMO-Journal Comp Hum Biol.

[13] Peterson MD, Saltarelli WA, Visich PS, Gordon PM (2014). Strength capacity and cardiometabolic risk clustering in adolescents. Pediatrics.

[14] Ekşioğlu M (2016). Normative static grip strength of population of Turkey, effects of various factors and a comparison with international norms. Appl Ergon.

[15] Ploegmakers JJW, Hepping AM, Geertzen JHB, Bulstra SK, Stevens M (2013). Grip strength is strongly associated with height, weight and gender in childhood: a cross sectional study of 2241 children and adolescents providing reference values. J Physiother.

[16] Shahida MSN, Zawiah MDS, Case K (2015). The relationship between anthropometry and hand grip strength among elderly Malaysians. Int J Ind Ergon.

[17] Chen C-Y, McGee CW, Rich TL, Prudente CN, Gillick BT (2018). Reference values of intrinsic muscle strength of the hand of adolescents and young adults. J Hand Ther.

[18] Omar MTA, Alghadir AH, Zafar H, Al Baker S (2018). Hand grip strength and dexterity function in children aged 6–12 years: A cross-sectional study. J Hand Ther.

[19] Björk M, Thyberg I, Haglund L, Skogh T (2006). Hand function in women and men with early rheumatoid arthritis. A prospective study over three years (the Swedish TIRA project). Scand J Rheumatol.

[20] Massy-Westropp NM, Gill TK, Taylor AW, Bohannon RW, Hill CL (2011). Hand grip strength: age and gender stratified normative data in a population-based study. BMC Res Notes.

[21] Cohen DD, Voss C, Taylor MJD, Stasinopoulos DM, Delextrat A, Sandercock GRH (2010). Handgrip strength in English schoolchildren. Acta Paediatr.

[22] Saremi M, Rostamzadeh S. Hand Dimensions and Grip Strength: A Comparison of Manual and Non-manual Workers. In: Congress of the International Ergonomics Association. Springer; 2018. p. 520–529.

[23] Rostamzadeh S, Saremi M, Tabatabaei S (2019). Normative hand grip strength and prediction models for Iranian office employees. Work.

[24] Rostamzadeh S, Saremi M, Taheri F (2020). Maximum handgrip strength as a function of type of work and hand-forearm dimensions. Work.

[25] Venckunas T, Emeljanovas A, Mieziene B, Volbekiene V (2017). Secular trends in physical fitness and body size in Lithuanian children and adolescents between 1992 and 2012. J Epidemiol Community Heal.

[26] Rostamzadeh S, Saremi M, Vahabzadeh-Monshi H, Yazdanparast P (2020). Grip and Pinch Strengths in Young Adults Residing in Tehran (2017): Development of Prediction Models. Iran J Heal Saf Environ.

[27] Wold S, Martens H, Wold H. The multivariate calibration problem in chemistry solved by the PLS method. In: Matrix pencils. Springer; 1983. p. 286–293.

[28] Reinartz W, Haenlein M, Henseler J (2009). An empirical comparison of the efficacy of covariance-based and variance-based SEM. Int J Res Mark.

[29] ISO 15535. 2012. General Requirements for Establishing Anthropometric Databases. In 2012.

[30] Kalton G, Brick JM, Lê T. Chapter VI Estimating components of design effects for use in sample design. 2005.

[31] Chen CW, Wang JY, Lou YT, Yeh YS, Tsai HL, Huang CW (2017). SUN-P087: The Prognostic Impact of Radiologic Assessment of Sacropenia and Osteopenia in Stage Iii Colon Cancer. Clin Nutr.

[32] Cruz-Jentoft AJ, Bahat G, Bauer J, Boirie Y, Bruyère O, Cederholm T (2018). Sarcopenia: revised European consensus on definition and diagnosis. Age Ageing.

[33] ISO 7250-1. 2017. Basic human body measurements for technological design -- Part 1: Body measurement definitions and landmarks. In 2017.

[34] Garrett JW (1971). The adult human hand: some anthropometric and biomechanical considerations. Hum Factors.

[35] Fess EE, Moran C. American society of hand therapists: Clinical assessment recommendations. Garner Soc. 1981. p 6–8.

[36] van den Beld WA, van den Beld WA, van der Sanden GAC, Sengers RCA, Verbeek ALM, Gabreëls FJM (2006). Validity and reproducibility of the Jamar dynamometer in children aged 4–11 years. Disabil Rehabil.

[37] Hébert LJ, Maltais DB, Lepage C, Saulnier J, Crête M, Perron M (2011). Isometric Muscle Strength in Youth Assessed by Hand-held Dynamometry: A Feasibility, Reliability, and Validity Study A Feasibility, Reliability, and Validity Study. Pediatr Phys Ther.

[38] Richards L, Palmiter-Thomas P. Grip strength measurement: a critical review of tools, methods, and clinical utility. Crit Rev Phys Rehabil Med. 1996;8(1–2).

[39] Hamilton A, Balnave R, Adams R (1994). Grip strength testing reliability. J Hand Ther.

[40] Bohannon RW (2017). Test-Retest Reliability of Measurements of Hand-Grip Strength Obtained by Dynamometry from Older Adults: A Systematic Review of Research in the PubMed Database. J frailty aging.

[41] Grubbs FE (1969). Procedures for detecting outlying observations in samples. Technometrics.

[42] Gefen D, Straub D, Boudreau M-C (2000). Structural equation modeling and regression: Guidelines for research practice. Commun Assoc Inf Syst.

[43] McDonald RP (1996). Path analysis with composite variables. Multivariate Behav Res.

[44] Chin WW, Marcolin BL, Newsted PR (2003). A partial least squares latent variable modeling approach for measuring interaction effects: Results from a Monte Carlo simulation study and an electronic-mail emotion/adoption study. Inf Syst Res.

[45] Hair JF Jr, Hult GTM, Ringle C, Sarstedt M. A primer on partial least squares structural equation modeling (PLS-SEM). Sage publications; 2016.

[46] Götz O, Liehr-Gobbers K, Krafft M. Evaluation of structural equation models using the partial least squares (PLS) approach. In: Handbook of partial least squares. Springer; 2010. p. 691–711.

[47] Selles RW, Zuidam JM, Willemsen SP, Stam HJ, Hovius SER (2010). Growth diagrams for grip strength in children. Clin Orthop Relat Res.

[48] Häger-Ross C, Rösblad B (2002). Norms for grip strength in children aged 4–16 years. Acta Paediatr.

[49] Ramos E, Frontera WR, Llopart A, Feliciano D (1998). Muscle strength and hormonal levels in adolescents: gender related differences. Int J Sports Med.

[50] Gómez-Campos R, Andruske CL, De Arruda M, Sulla-Torres J, Pacheco-Carrillo J, Urra-Albornoz C (2018). Normative data for handgrip strength in children and adolescents in the Maule Region, Chile: Evaluation based on chronological and biological age. PLoS One.

[51] Omar MTA, Alghadir A, Al Baker S (2015). Norms for hand grip strength in children aged 6–12 years in Saudi Arabia. Dev Neurorehabil.

[52] de Souza MA, Benedicto MMB, Pizzato TM, Mattiello-Sverzut AC (2014). Normative data for hand grip strength in healthy children measured with a bulb dynamometer: a cross-sectional study. Physiotherapy.

[53] Celis-Morales CA, Petermann F, Steell L, Anderson J, Welsh P, Mackay DF (2018). Associations of dietary protein intake with fat-free mass and grip strength: a cross-sectional study in 146,816 UK Biobank participants. Am J Epidemiol.

[54] Heath B, Shelton M, Stief C, Summerson S. Handgrip Strength Positively Correlates With Percent Fat Free Mass in Students at Messiah College. In: International Journal of Exercise Science: Conference Proceedings. 2019. p. 42.

[55] Round JM, Jones DA, Honour JW, Nevill AM (1999). Hormonal factors in the development of differences in strength between boys and girls during adolescence: a longitudinal study. Ann Hum Biol.

[56] Dore E, Martin R, Ratel S, Duché P, Bedu M, Van Praagh E (2005). Gender differences in peak muscle performance during growth. Int J Sports Med.

[57] Dempsey PG, Ayoub MM (1996). The influence of gender, grasp type, pinch width and wrist position on sustained pinch strength. Int J Ind Ergon.

[58] Sartorio A, Lafortuna CL, Pogliaghi S, Trecate L (2002). The impact of gender, body dimension and body composition on hand-grip strength in healthy children. J Endocrinol Invest.

[59] Ng AK, Hairi NN, Jalaludin MY, Majid HA (2019). Dietary intake, physical activity and muscle strength among adolescents: the Malaysian Health and Adolescents Longitudinal Research Team (MyHeART) study. BMJ Open.

[60] Rauch F, Neu CM, Wassmer G, Beck B, Rieger-Wettengl G, Rietschel E (2002). Muscle analysis by measurement of maximal isometric grip force: new reference data and clinical applications in pediatrics. Pediatr Res.

[61] Luna-Heredia E, Martín-Peña G, Ruiz-Galiana J (2005). Handgrip dynamometry in healthy adults. Clin Nutr.

[62] Cortell-Tormo JM, Pérez Turpin JA, Lucas Cuevas ÁG, Pérez-Soriano P, Llana Belloch S, Martínez Patiño MJ (2013). Handgrip strength and hand dimensions in high-level inter-university judoists..

[63] Golub MS, Collman GW, Foster PMD, Kimmel CA, Rajpert-De Meyts E, Reiter EO (2008). Public health implications of altered puberty timing. Pediatrics.

[64] Boadella JM, Kuijer PP, Sluiter JK, Frings-Dresen MH (2005). Effect of self-selected handgrip position on maximal handgrip strength. Arch Phys Med Rehabil.

[65] Maleki-Ghahfarokhi A, Dianat I, Feizi H, Asghari-Jafarabadi M (2019). Influences of gender, hand dominance, and anthropometric characteristics on different types of pinch strength: A partial least squares (PLS) approach. Appl Ergon.

[66] Rostamzadeh S, Saremi M, Bradtmiller B (2020). Age, gender and side-stratified grip strength norms and related socio-demographic factors for 20–80 years Iranian healthy population: Comparison with consolidated and international norms. Int J Ind Ergon.

[67] Nicolay CW, Walker AL (2005). Grip strength and endurance: Influences of anthropometric variation, hand dominance, and gender. Int J Ind Ergon.

[68] Bohannon RW, Wang Y-C, Bubela D, Gershon RC (2017). Handgrip strength: a population-based study of norms and age trajectories for 3-to 17-year-olds. Pediatr Phys Ther.

[69] Parker DF, Round JM, Sacco P, Jones DA (1990). A cross-sectional survey of upper and lower limb strength in boys and girls during childhood and adolescence. Ann Hum Biol.

